# Insights: fear of GAD

**DOI:** 10.1002/2211-5463.13104

**Published:** 2021-02-11

**Authors:** Duncan Wright

**Affiliations:** ^1^ FEBS Open Bio Editorial Office Cambridge UK

## Abstract

Welcome to *Insights*, a new series in which articles published in *FEBS Open Bio* are summarised for the wider community. We hope that this series will help make the findings we publish more accessible to the general public and encourage greater engagement. In this first article of the series, we introduce a research paper on fear in rats, authored by Professor Yuchio Yanagawa and colleagues and published in this issue. Photo: Young rat and cat in front of black background. Cynoclub/Shutterstock.com.

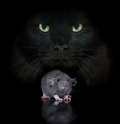

Welcome to *Insights*, a new series in which articles published in *FEBS Open Bio* are summarised for the wider community. We hope that this series will help make the findings we publish more accessible to the general public and encourage greater engagement. In this first article of the series, we introduce a research paper on fear in rats, authored by Professor Yuchio Yanagawa and colleagues and published in this issue.

Anxiety, a symptom of many psychiatric disorders, is not only detrimental to the individual’s quality of life, but also places a heavy burden on friends, relatives, mental health services and the economy [1]. While both psychological and pharmacological therapies are available for anxiety, these have associated risks (such as side effects and dependence after long‐term use) and may not be effective in all cases [2]. A greater understanding of how the brain normally regulates fear would help researchers develop more selective treatments with reduced adverse effects.

Our nerve cells, or neurons, communicate with each other using various small molecules, known as neurotransmitters. One of the main neurotransmitters in the brain and spinal cord is called γ‐aminobutyric acid, or GABA, and this molecule is known to be important for the control of fear in the amygdala [3]. Our bodies produce GABA using an enzyme called glutamic acid decarboxylase, or GAD [3]. Two different genes encode slightly different versions of GAD: a larger form, called GAD67, and a smaller form, called GAD65 [3]. Levels of the larger GAD67 protein are decreased in the brains of patients with schizophrenia and major depressive disorder [4], but this does not imply that decreased GAD67 causes these conditions; it is possible that these conditions and decreased GAD67 are both caused by some other factor.

One way to investigate the role of an enzyme is to generate mutant mice which lack it and then examine their behaviour. This strategy worked for the shorter GAD65 enzyme: mice without a functional copy of GAD65 exhibit increased anxiety‐like behaviour and an increase in generalised fear (i.e. the animals are more likely to associate fear of one stimulus with a second, related stimulus) [5, 6]. Unfortunately, mice lacking GAD67 die soon after birth and so the role of this enzyme in fear and anxiety is less well understood than that of GAD65 [7].

Previously, technical limitations largely restricted genetic engineering to certain well‐studied model organisms, such as mice. However, a recently developed technique, called CRISPR, has enabled researchers to easily (relatively speaking…) modify gene sequences in various organisms, including rats [8]. A team of researchers led by Professor Yuchio Yanagawa at Gunma University, Japan, recently took advantage of this technique to see if the effect of GAD67 could be studied in rats instead of mice. To their surprise, about 33% of mutant rats lacking GAD67 survived to adulthood [9].

In this issue of *FEBS Open Bio*, Professor Yanagawa and colleagues used their newly generated rats to examine how the absence of GAD67 affects fear behaviour [4]. Fear can be quantified by measuring the amount of time rats exhibit a ‘freezing’ response – that is, freeze without moving. The researchers performed Pavlovian fear conditioning to determine whether the absence of GAD67 affected the ability of rats to learn to associate certain events (such as the test cage or a tone with a particular frequency) with a mild electric shock. The mutant rats exhibited increased and unusual patterns of freezing as compared to control rats, suggesting that the lack of GAD67 enhances fear responses.

To ensure that any responses observed are related to fear, the researchers were careful to test for potential confounding factors. For example, if the lack of GAD67 affected hearing, this would reduce the ability of the rats to associate a tone with the electric shock. The authors confirmed that both hearing and sensitivity to the shock were not affected in rats lacking GAD67. However, the mutant rats were found to spend less time moving around an enclosure than control rats, and this general reduction in movement might be expected to influence the performance of the rats in the memory tests. After taking this variation into account, the authors concluded that the increased freezing behaviour of the mutant rats was likely due to increased anxiety, and not due to reduced locomotory activity.

The current findings may also have significance beyond understanding the role of GAD and GABA in fear: last year, two studies described patients lacking functional GAD67 who presented with epilepsy and developmental defects, including cognitive impairments [10, 11]. On this basis, Professor Yanagawa and colleagues suggest that rats lacking GAD67 may exhibit more general memory defects that affect their ability to associate the tone with the electric shock [4]. Teasing apart any such effect on memory will require further characterisation of the rats. In addition, as the current findings on GAD67 were obtained using rats and the earlier findings on GAD65 with mice, the effects of these two enzymes cannot be directly compared at present. Future studies on rats lacking GAD65 will provide a more complete picture of the respective roles of these enzymes in fear and anxiety.
